# Federated Learning Approach with Pre-Trained Deep Learning Models for COVID-19 Detection from Unsegmented CT images

**DOI:** 10.3390/life12070958

**Published:** 2022-06-26

**Authors:** Lucian Mihai Florescu, Costin Teodor Streba, Mircea-Sebastian Şerbănescu, Mădălin Mămuleanu, Dan Nicolae Florescu, Rossy Vlăduţ Teică, Raluca Elena Nica, Ioana Andreea Gheonea

**Affiliations:** 1 Department of Radiology and Medical Imaging, University of Medicine and Pharmacy of Craiova, 200349 Craiova, Romania; lucian.florescu@umfcv.ro (L.M.F.); ioana.gheonea@umfcv.ro (I.A.G.); 2 Department of Pneumology, University of Medicine and Pharmacy of Craiova, 200349 Craiova, Romania; costin.streba@umfcv.ro; 3 Department of Medical Informatics and Biostatistics, University of Medicine and Pharmacy of Craiova, 200349 Craiova, Romania; mircea.serbanescu@umfcv.ro; 4 Department of Automatic Control and Electronics, University of Craiova, 200585 Craiova, Romania; 5 Department of Gastroenterology, University of Medicine and Pharmacy of Craiova, 200349 Craiova, Romania; 6 Doctoral School, University of Medicine and Pharmacy of Craiova, 200349 Craiova, Romania; rossy.teica@gmail.com (R.V.T.); raluca.elena.nica@gmail.com (R.E.N.)

**Keywords:** federated learning, COVID-19, computed tomography

## Abstract

(1) Background: Coronavirus disease 2019 (COVID-19) is an infectious disease caused by SARS-CoV-2. Reverse transcription polymerase chain reaction (RT-PCR) remains the current gold standard for detecting SARS-CoV-2 infections in nasopharyngeal swabs. In Romania, the first reported patient to have contracted COVID-19 was officially declared on 26 February 2020. (2) Methods: This study proposes a federated learning approach with pre-trained deep learning models for COVID-19 detection. Three clients were locally deployed with their own dataset. The goal of the clients was to collaborate in order to obtain a global model without sharing samples from the dataset. The algorithm we developed was connected to our internal picture archiving and communication system and, after running backwards, it encountered chest CT changes suggestive for COVID-19 in a patient investigated in our medical imaging department on the 28 January 2020. (4) Conclusions: Based on our results, we recommend using an automated AI-assisted software in order to detect COVID-19 based on the lung imaging changes as an adjuvant diagnostic method to the current gold standard (RT-PCR) in order to greatly enhance the management of these patients and also limit the spread of the disease, not only to the general population but also to healthcare professionals.

## 1. Introduction

Coronavirus disease 2019 (COVID-19) is an infectious and highly contagious viral disease caused by severe acute respiratory syndrome coronavirus 2 (SARS-CoV-2). This disease has been responsible for extreme global lockdown measures, millions of deaths, and major socioeconomic havoc [1]. COVID-19 is thought to have been identified in Wuhan, China in late 2019 before rapidly spreading on a global scale and developing into a pandemic on 11 March 2020, according to the World Health Organization (WHO) [2]. As of May 2022, the disease has affected more than 520 million and killed over 6.2 million people worldwide. With more than 500,000 new cases and thousands of deaths recorded on a daily basis around the world, this pandemic appears to be far from over and seems like an exhausting and everlasting battle between a continuous emergence of new viral strains and an ongoing development of new vaccines and antiviral treatments [3].

The infection with SARS-CoV-2 can either remain asymptomatic or lead to the development of non-specific signs and symptoms such as fever, cough, partial/complete loss of smell and/or taste, fatigue, and shortness of breath in some individuals [4,5,6]. However, little information still remains known about the long-term effects of this disease on individual health. The main medical imaging methods used to detect COVID-19 are chest X-ray and computed tomography (CT), with the latter being vastly superior in terms of accuracy (ACC). The typical lung CT changes detected in COVID-19 patients include ground-glass opacities ± lung consolidation areas usually distributed in the posterior and peripheral regions of both lungs, frequently affecting multiple lobes [7,8].

Reverse transcription-polymerase chain reaction (RT-PCR) still remains the current gold standard for detecting this viral infection in nasopharyngeal swabs. This diagnostic method features high sensitivity (Sen) (up to 97.8%) and high specificity (Spe) (up to 100%) [9]. Artificial intelligence (AI)-based software has largely been used in many domains that require pattern or image recognition and classification, thus leading to multiple applications in order to improve almost every aspect of our lives such as self-driving cars, facial recognition, personalized virtual shopping assistants, marketing, and financial robots, etc. One particular application of AI-based software is in medicine where the ability to recognize different patterns or image characteristics leads to a novel way to detect various diseases based on the histopathology and/or radio-imaging aspect [10]. In search for an adjuvant COVID-19 diagnostic method, an independent AI-based software previously trained on a large set of chest CT scans may provide good Sen and Spe in detecting pulmonary changes caused by this disease [11].

This study aims to develop a large image dataset containing unsegmented axial chest CT images in lung window in order to perform a differential diagnosis (solely based on the chest CT imaging aspect) between normal lung aspect, COVID-19, non-COVID-19 lung infections, and lung cancer by using a federated learning (FL) approach with pre-trained deep learning (DL) models for COVID-19 detection. Moreover, our automated AI software is able to connect to an internal picture archiving and communication system (PACS) and analyze previous CT scans in order to identify COVID-19 patients prior to the officially declared patient zero in each region, solely based on the chest CT changes.

## 2. Related Works

Ever since the pandemic started in the first half of 2020, there have been numerous attempts to develop an AI-assisted software that is capable of identifying COVID-19 based solely on the chest CT imaging findings. Given the global spread of COVID-19, machine learning (ML) and DL approaches have been used in order to diagnose this disease on both X-ray and chest CT [12]. Algorithms based on DL allow computational models to have an increased ACC in classifying different objects or sounds in order to provide a superior output compared to humans in various scenarios [13,14].

### 2.1. Detecting Lung Diseases on X-ray Images Using Convolutional Neural Networks (CNNs)

Vieira et al. made use of several CNNs (i.e., DenseNet201, IncepetionResNetV2, InceptionV3, NASNetLarge, ResNet50, VGG16, and Xception) for classifying chest X-ray images and published one of the most recent and prominent papers in this field. The authors of the study used frontal chest X-rays classified into five different image datasets. The COVID-19 dataset was named ‘COVID-DB’, consisted of three datasets, and included 717 images. Another dataset named ‘NIH Chest-X-ray’ included 61,754 X-rays and was made publicly available by the National Health Institute (60,412 normal aspect, 1342 pneumonia with/without comorbidities). The last dataset consisted of 5863 frontal chest X-rays (2782 bacterial pneumonia, 1583 normal aspect, and 1493 viral pneumonia) and was used for testing the ACC of the algorithm. The highest ACC was obtained using ResNet50 (0.990 ± 0.003) and VGG16 (0.990 ± 0.004). The highest Sen was achieved using VGG16 (0.981 ± 0.022), while the highest Spe was obtained using ResNet50 (0.996 ± 0.003) [15].

Aslan et al. proposed a different approach to detect COVID-19 infection in individuals by using a CNN-based transfer learning (TL): BiLSTM network. The study required a large image database which consisted of 2905 chest X-rays in total (219—COVID-19, 1345—viral pneumonia, 1341—normal lung aspect). The data available initially in the dataset was enhanced using lung segmentation and data augmentation in order to increase classification success. The authors developed a modified AlexNet network (mAlexNet) by adjusting the last three layers in order to classify X-ray images as normal lung aspect, viral pneumonia, or COVID-19. When using the mAlexNet architecture, the model obtained a 98.14% ACC. However, when combined with BiLSTM, the ACC increased slightly (98.70%) [16].

Apostolopoulos et al. used TL combined with CNNs in order to automatically detect COVID-19 changes on X-ray images. Two datasets were developed: Dataset_1—1428 X-rays (224 images with confirmed COVID-19, 700 images with confirmed common bacterial pneumonia, 504 images with normal lung aspect) and Dataset_2—1442 X-rays (224 images with confirmed COVID-19, 714 images with both bacterial and viral pneumonia and 504 with normal lung aspect). The authors used multiple networks for TL (VGG19, MobileNet v2, Inception, Xception, Inception ResNet v2) and achieved the highest ACC (98.75%) when detecting COVID-19 alone when using VGG19. Also, VGG19 was able to correctly classify X-rays as normal, pneumonia, or COVID-19 with a recorded ACC of 93.48% [17].

Several different approaches using CNNs to detect COVID-19 on chest X-rays are presented in Table 1.

### 2.2. Detecting Lung Diseases on Chest CT Scans Using CNNs

Ardakani et al. made use of DL techniques in order to detect COVID-19 based on CT images. The study included a total of 1020 images (510—non-COVID-19 and 510—COVID-19) out of which 816 were used for training, and the rest were used for validation. The authors used ten CNNs (AlexNet, GoogLeNet, VGG-16, VGG-19, SqueezeNet, MobileNet-V2, ResNet-18, ResNet-50, ResNet-101, Xception) to train and validate the datasets. The highest performance in both training and validation was achieved using Xception (Sen: 98.77%; Spe: 100%; ACC: 99.38%) and ResNet-101 (Sen: 100%; Spe: 99.26%; ACC: 99.63%) [26].

Ahuja et al. developed a three-phased methodology for detecting COVID-19 on chest CT scans: data augmentation, TL, and abnormality detection using a deeper layer. The used dataset consisted of 349 chest CT images of COVID-19 patients and 397 chest CT images illustrating a non-COVID-19 aspect. The tested TL models were ResNet18, ResNet50, ResNet101, SqueezeNet. The best performing model was Res-Net18, which achieved a training ACC of 99.82%, a validation accuracy of 97.32%, and a testing ACC of 99.4%. When classifying COVID-19 and non-COVID-19 chest CT images, this methodology achieved 98.6% specificity and 100% sensitivity, reaching an area under the curve (AUC) of 0.9965 [27].

Several different approaches using CNNs to detect COVID-19 on chest CT scans are presented in Table 2.

While these centralized approaches of using CNN for detecting COVID-19 in either X-ray or CT images have been proven to be accurate, they have a major drawback. In practice, a collaboration between medical institutions is difficult due to privacy concerns. Thus, building a dataset with enough samples to train a DL model can be difficult.

Ines Feki et al. [33] proposed a FL approach for COVID-19 detection in X-ray images. In their work, VGG-16 and ResNet50 [34] pretrained models were used. X-ray images were used as inputs in the CNN models and divided the dataset into four subsets, each subsets belonging to a FL client. The obtained model in their work was a binary classifier predicting whether an X-ray image is from a patient with COVID-19 or not. In their work, Rajesh Kumar et al. [35] proposed blockchain FL for COVID-19 detection in CT images. Their approach was to perform image segmentation on the CT images by using SegCaps [36] and then train a capsule network [37] using the outputs from the SegCaps network. As mentioned for [33], the obtained model was a binary classifier. In their work [38], Boyi Liu et al. experimented with four different CNN models for detecting COVID-19 (COVID-Net, MobileNet_v2, ResNet18 and ResNeXt) in X-ray images by using FL. Their conclusion was that ResNeXt and ResNet18 were a better choice in term of COVID-19 identification in X-ray images.

## 3. Materials and Methods

### 3.1. Materials

The image dataset developed in this study included a total of 2230 axial chest CT images in lung window and was further divided into three groups: (a) COVID-19 (1016 images), (b) lung cancer, and non-COVID-19 lung infections (610 images), and (c) normal lung aspect (604 images).

The COVID-19 image database was built using chest CT scans from the internal PACS of the Medical Imaging Department of the University of Medicine and Pharmacy of Craiova, in addition to other reliable free online access public image datasets: Radiopaedia [4], Radiology Assistant [7], Harvard Dataverse (a COVID-19 CT Dataset with Open-Access chest CT images of over 1000 patients with confirmed COVID-19 diagnosis) [39], and the COVID-19 common pneumonia chest CT dataset (416 COVID-19 positive CT scans) [40].

The lung cancer and non-COVID-19 lung infections image dataset was developed using multiple reliable free online access public chest CT examinations: Radiopaedia (pneumonia [41], lung abscess [42], lung hydatid infection [43], tuberculosis [44], primary lung cancer [45], pulmonary metastases [46]) and the COVID-19 and common pneumonia chest CT dataset (412 common pneumonia CT scans) [47]. Morever, this dataset also included a large-scale CT and PET/CT dataset for lung cancer diagnosis (Lung-PET-CT-Dx) [48], as well as chest CT scans from the internal PACS of the Medical Imaging Department at the University of Medicine and Pharmacy of Craiova.

The normal lung aspect image database was entirely developed using chest CT scans from the internal PACS of the Medical Imaging Department at the University of Medicine and Pharmacy of Craiova.

All images classified as COVID-19 came from patients with a positive RT-PCR result. For patients confirmed with COVID-19, chest CT scans were performed within 1 to 10 days from the RT-PCR test, in order to boost the detection of COVID-19 chest CT pathologic changes over the course of the disease. All patient data was anonymized, and the study was approved by the Ethical Board of the University of Medicine and Pharmacy of Craiova (no. 101/20.05.2022).

### 3.2. Methods

#### 3.2.1. TL

Artificial neural networks, also known as neural networks (NNs), are computing systems inspired by the biological neural networks based on a collection of connected computing units (nodes) called artificial neurons [49]. They have revolutionized almost all medical fields, showing better results than other ML approaches [50,51]. It has been proven that a NN would only require two hidden layers of nodes in order to converge on any dataset [52].

The DL concept refers to networks that have more intermediate layers, and in the area of image processing, they usually contain some convolutional layers [53] that make use of kernels (also known as filters) that slide along input features and provide translation equivalent responses known as feature maps [54]. TL implies converting a network that was trained for a specific job to respond to a new one, and this happens by replacing some existing layers (usually the last layers) and keeping the rest of the layers together with the weights. The new architecture goes through a learning process again, and the results are usually better [55].

#### 3.2.2. FL and Model Description

When training a DL model, the process usually happens on a central server or workstation. For the training process, the dataset must be stored on that specific machine, or an external storage provider must be mounted. While this type of process has been proven to be highly effective for training DL models, in some cases, in which the dataset is comprised of data from multiple sources, this type of training process cannot be done due to both legal and privacy concerns. FL [56] is a process for training AI models. The training is performed on multiple machines and each of these machines has its own dataset. Each of the machines involved in the training process uploads to the central server the updated weights without disclosing the dataset on which the model was trained. FL technique applies only to supervised learning. In contrast with the conventional training process described earlier, in FL, no samples from the dataset are uploaded to the central server. FL techniques for training a DL model can be divided into two categories: centralized and decentralized. For centralized FL, a central server is involved in order to manage all the steps performed by each machine and to coordinate each machine in the training process [57]. Moreover, the central server is the one in charge to query the available devices and request the updated weights from each machine. In decentralized FL, the central server is no longer needed, and each machine involved in the training process coordinates to create a global model [57]. The model is then sent to all the machines in the network. Decentralized FL is also created using blockchain [58]. 

For the proposed method, a centralized FL technique was used. The proposed architecture contained a central server and three clients. Each client had its own dataset batch. The assumptions made in our method were that there were three separate institutions with three datasets and a central server that had to compile a global model for all three institutions without sharing the dataset between them. To eliminate network latency that can impact the FL process [57], all three nodes were deployed on the same physical machine. As mentioned earlier, when FL was performed, the central server did not have access to the datasets each node was trained on. Each node had sent the updated weights to the central server. For compiling the global model, the federated averaging algorithm (FedAvg) was used [59]. FedAvg algorithm is given by Equation (1), in which k is the total number of clients that participated in a specific training round, t represents the training rounds and w^k^ represents the weights updated by client k.

(1) $$w^{t+1}=\frac{\sum_{i=1}^{k}w_{i}^{t}}{k}$$

**Figure 1:**
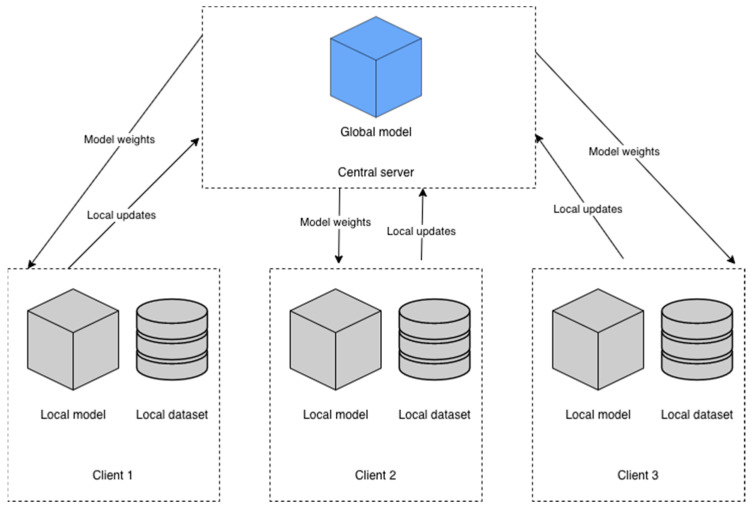
Diagram of the proposed system.

FL is an iterative process, in which the central server is sending the initial model to the clients, and the clients train the model with their data and send updates to the central server. A FL round is a process in which all these steps are performed. Each client has its own model with the corresponding weights. For the proposed study, a client contained a VGG-16 model [60]. Since this model was very large and required a large amount of data to train, TL techniques were used. The weights loaded into each model for each FL entity were obtained from training the model on the ImageNet dataset [61]. ImageNet dataset contained around 14 million labeled images with 20,000 classes. VGG-16 model is a CNN classifier proposed by Karen Simonyan et al. [60], which has an architecture with small convolutional filters (3 × 3). Karen Simonyan et al. [60] proposed the VGG-16 architecture with different layer configurations. All VGG-16 configurations have a stack of convolutional layers and a stack of fully connected layers. Depending on the configuration, a VGG-16 model can have between 133 and 144 million parameters [60]. For all configurations of VGG-16, the input is a 224 × 224 × 3 tensor. The image is passed through a stack of convolutional and fully connected layers (the number varies depending on configuration). Each configuration followed a similar structure, the difference being the number of layers in each configuration: 11 layers (8 convolutional and 3 fully connected layers) in A, 13 layers (10 convolutional and 3 fully connected layers) in B configuration, 16 layers in C and D configuration and 19 layers in E configuration. For the proposed method, the D model configuration was chosen. The configuration is presented in Table 3. For this model, the input tensor was not changed: 224 by 224 with 3 channels. VGG-16 was chosen as it has been proven to be accurate in classifying X-ray and CT images [23,25]. Moreover, in order to locally deploy three FL clients, a model that could fit three times on the GPU was needed. For the proposed model distributed to each client, the fully connected layer proposed in [60] was dropped and replaced with a fully connected layer with 128 neurons with a rectified linear unit as the activation function, followed by a fully connected layer with three neurons, with Softmax as activation function (Equation (3)). Thus, the model was processing the input tensor and provided as an output a vector with three values as presented in Equation (2). Each value from the vector represented the probability for each class. The diagram of the proposed method is presented in Figure 1.

(2) $$\hat{y}=\left[\begin{array}{c} \hat{y_{1}}\\ \hat{y_{2}}\\ \hat{y_{3}} \end{array}\right]$$

(3) $$\sigma \left(\vec{z}\right)=\frac{e^{z_{i}}}{\sum_{j=1}^{K}e^{z_{j}}}$$

where $\vec{z}$ is the input vector and K is the number of classes.

The loss function used for the VGG-16 models deployed on each entity was cross-entropy. Cross entropy is a loss function that measures the performance of a classification model. In applications where there are two classes, the cross-entropy function becomes binary cross-entropy. However, the proposed method is a multiclass classification. Therefore, for calculating the loss of each model for each entity deployed, a separate loss was computed for each label and for each observation. The final loss for each entity involved in the training and validation process was computed as the sum of the losses for each label (Equation (4)). Where L was the number of classes, y was the binary value indicating if observation o was correcting classifying the label i, and p was the predicted probability of observation o for class i.

(4) $$-\sum_{i=1}^{L}y_{o,i}\log\left(p_{o,i}\right)$$

#### 3.2.3. Hyperparameters and FL Configuration

For the proposed method, two categories of parameters can be defined. The first category is represented by the parameters that are configured for each VGG-16 DL model deployed on each client. When performing training of a DL model, the values computed by the loss function are indicating whether a model is converging or not. These values are passed to the optimizer of the DL model to decide what weights to update. The optimizer chosen for the proposed model was Adam [62]. The Adam optimizer is based on the stochastic gradient descent (SGD) algorithm. However, unlike SGD which keeps the learning rate constant for all weights updated, Adam used a learning rate for each parameter. By having a learning rate for each parameter, Adam improved the performance in computer vision tasks, especially in sparse or noisy gradients [62]. Adam introduced four configuration parameters. Alpha (α), the learning rate, a parameter that indicated the step size at each iteration. Beta1 and Beta2 (β_1_, β_2_), the exponential decay rates in which β_1_, β_2_ ∈ [0, 1). Epsilon (ε), a small value for preventing division by zero. The values recommended by Diederik P.K. et al. [62] were used: α = 0.001, β_1_ = 0.9, β_2_ = 0.999 and ε = 10^−8^. Adam was used as an optimizer for all three clients. For each client, the chosen batch size was 50 and 15 epochs. For the FL configuration, a total number of 10 training rounds were chosen.

The labels from the dataset were encoded as follows: 0 for COVID-19, 1 for non-COVID-19 pneumonia and lung cancer, and 2 for normal lung aspect. After this step, the images were placed in three different folders named according to the integer encoded number. These paths on the local machine were parsed into three mutable lists, a list of images for each disease. To randomly divide the dataset for the three clients, each list was shuffled and divided into three sub-lists. After this step, each client had its own unique dataset. The proposed method first initialized a global model by loading the weights of the pre-trained VGG-16 model. This step was performed by the central server. Then, the central server randomly selected N clients from the available client list. In our method, N was equal to two, meaning that if two out of the three clients were available, the training or evaluation could start. The central server then distributed the weights downloaded at the beginning of the procedure to each client. Finally, each client performed the training and sent the updated weights to the central server. The central server aggregated a new model based on FedAvg [59] given by Equation (1).

## 4. Results

For evaluating the performance of the aggregated model, the following metrics were observed: categorical accuracy, F1-score micro, F1-score macro, Cohen’s kappa score, and Matthews correlation coefficient. These metrics were defined by the following equations:

(5) $$Categorical\;Accuracy=\frac{n}{N}$$

(6) $$F1_{\mu }=\frac{2\cdot Precision_{\mu }\cdot Recall_{\mu }}{Precision_{\mu }\cdot Recall_{\mu }}$$

(7) $$F1_{M}=\frac{2\cdot Precision_{M}\cdot Recall_{M}}{Precision_{M}\cdot Recall_{M}}$$

where n represented the number of correct predictions, and N represented total predictions. Indices μ and M indicated micro-averaging and macro-averaging, respectively. In binary classification, the F1 score is computed as a harmonic mean between precision and recall. For the proposed method, which represents a multiclass model, micro-averaged and macro-averaged precision and recall were used to compute F1-micro and F1-macro. Precision-micro, precision-macro, recall-micro, and recall macro are given by Equations (8)–(11), where TP is true positives, FP is false positives, FN is false negatives, and k is the total number of classes.

(8) $$Precision_{\mu }=\frac{\sum_{i=1}^{k}TP_{i}}{\sum_{i=1}^{k}\left(TP_{i}+FP_{i}\right)}$$

(9) $$Precision_{M}=\frac{\sum_{i=1}^{k}\frac{TP_{i}}{TP_{i}+FP_{i}}}{k}$$

(10) $$Recall_{\mu }=\frac{\sum_{i=1}^{k}TP_{i}}{\sum_{i=1}^{k}\left(TP_{i}+FN_{i}\right)}$$

(11) $$Recall_{M}=\frac{\sum_{i=1}^{k}\frac{TP_{i}}{TP_{i}+FN_{i}}}{k}$$

Cohen’s kappa score is a performance metric that is used to assess the agreement between two parties. For ML or DL models, Cohen’s kappa score is used to compare the predictions of a model with the actual values. Cohen’s kappa score is defined by Equation (12) [63], in which p_0_ is the observed agreement and p_e_ is the expected agreement. The value of k should be less than or equal to 1. Besides measuring the performance of a DL model, Cohen’s k score can be used to compare two models which have similar accuracy.

(12) $$kk=\frac{p_{0}-p_{e}}{1-p_{e}}$$

The Matthews correlation coefficient (MCC) is a performance metric used in ML or DL which can measure the quality of a binary classifier. Thus, for a binary classifier, MCC is given by Equation (13). However, the proposed study contained a multiclass DL classifier. MCC was generalized for multiclass classification [64] and for a K-by-K confusion matrix, is given by Equation (14), where c is the correctly predicted samples, s is the total number of samples, p_k_ is the number of predictions for class k, and t_k_ denotes the number of occurrences for class k.

(13) $$MCC=\frac{TP\times TN-FP\times FN}{\sqrt{\left(TP+FP\right)\left(TP+FN\right)\left(TN+FP\right)\left(TN+FN\right)}}$$

(14) $$MCC=\frac{c\times s-\sum_{k}^{K}p_{k}\times t_{k}}{\sqrt{(s^{2}-\sum_{k}^{K}p_{k}^{2})\;\left(s^{2}-\sum_{k}^{K}t_{k}^{2}\right)}}$$

For comparison purposes only, the same pre-trained DL model, VGG-16, was trained in a centralized way on all images in the dataset and the same metrics were observed: categorical accuracy, F1-score micro, F1-score macro, Cohen’s kappa score, and MCC. For visualizing the final convolutional layer and producing a localization map for the important regions in the images, gradient-weighted class activation map (Grad-CAM) was used [65]. The results obtained for centralized VGG-16 and FL VGG-16 models during training are presented in Table 4. The performance of each model during the validation stage is presented in Table 5. As an additional step, the training time in seconds for the FL approach and centralized approach was added in Table 4. All the implementations were done using Python programming language, version 3.7. For training the models, Tensorflow version 2.8.0 was used [66] with Keras version 2.4.0. The FL framework used for this study was Flower.dev [67] version 0.18.0. All the FL clients were deployed on the same machine, equipped with an Intel Xeon processor, 128GB of RAM, and an Nvidia Quadro RTX 6000 GPU.

**Figure 2:**
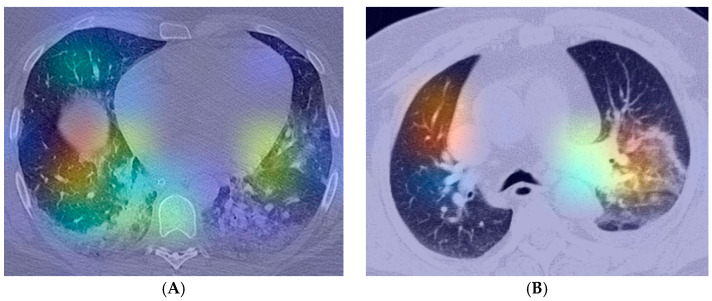
Class activation map—(**A**) COVID-19; (**B**) lung cancer or other non-COVID-19 infections.

In Figure 2, Grad-CAMs for two randomly selected images in the dataset (COVID-19 and Lung cancer or other non-COVID-19 infections labels) are presented. The images were obtained by displaying the output of the layer with the Id 5 as presented in Table 3. The Grad-CAM for the two images indicated that, for feature extraction, the proposed model is focusing on extracting features from the lung lesions. It is important to note that the algorithm detects only lung lesions without highlighting false positive changes in the mediastinal structures.

## 5. Discussion

The approach presented in our paper involves using a medical imaging method (chest CT) in detecting COVID-19 as an automated adjuvant diagnostic method to the current gold standard (RT-PCR). Compared to RT-PCR, CT devices are widely available worldwide, are able to assess patient prognosis in the course of the disease, can rapidly provide a test result, and greatly enhance the daily testing capacity. The DL model was obtained as an aggregated model from a FL training. Our proposed method can be used as an unsupervised and completely independent software that performs a differential diagnosis (solely based on CT imaging aspect) between normal lung aspect, COVID-19, and lung cancer and non-COVID-19 lung infections. Moreover, since the three clients involved in the training process did not share any samples from the dataset, our method can be used as a collaborative technique between medical institutions to build a common DL model for COVID-19 detection without sharing private data.

Compared to other AI-based applications that detect COVID-19 using chest CT scans, our solution not only detects lung signs of SARS-CoV-2 infection but also indicates alternative diagnostics in case of a negative test result.

This paper proposed a FL approach for COVID-19 detection with pre-trained DL models. FL is a process for training AI models. The training can be performed on multiple machines and each of these machines has its own dataset. A central server coordinates all machines with the goal of obtaining a global model. When the model is obtained, the weights of the model are sent back to each machine in order to update the model stored locally. In the proposed method, three FL entities were deployed locally, each with a pretrained VGG-16 [60] model. The configuration chosen for this model was D-configuration as presented in Table 3 [60]. The fully connected layers of each VGG-16 were dropped and replaced with a fully connected layer with 128 neurons with Rectified linear unit as activation function followed by a fully connected layer with 3 neurons with Softmax as activation function. Convolutional layers of the DL model were frozen in order to prevent these from modifying during training. The aggregated model is a multiclass model, having three neurons as output: COVID-19, cancer, and non-COVID-19 lung infections or normal lung. To properly assess the performance of the aggregated model, categorical accuracy, F1_μ_, F1_M_, Cohen’s Kappa score, and MCC were observed. Categorical accuracy represents the percentage of predicted classes that match with the actual labels. While categorical accuracy alone cannot provide complete information about the performance of the model, it shows that the aggregated model is classifying correctly 83.82% of the images during training and 79.32% during validation. In [35], experiments in a blockchain-federated learning configuration with different classifiers were performed. The inputs of the classifiers were CT images. Except for one, all of the classifiers were pretrained on ImageNet dataset. Their results show a precision of 0.8269 with specificity of 0.1561 and sensitivity of 0.8294 for VGG-16 model. However, the model proposed by Rajesh Kumar et al. [35] is a binary classifier. In their work, Ines Feki et al. [33] proposed a FL approach for COVID-19 classification in chest X-ray images. While the FL VGG-16 model obtained in [33] performed well with 93.57 accuracy and 92.12 specificity, the authors observed that the performance of the model was significantly affected when one or more clients are not involved in the training process.

The F1 score is defined as a harmonic mean between precision and recall. The precision metric indicates if the aggregated model is accurate in classifying dataset samples as positive while the Recall metric indicates if the positive samples are classified correctly without taking into account the false positives. The macro average of the F1 score is the arithmetic mean of the F1 score for each label. Thus, all classes have the same weight. From Table 4, it can be observed that in terms of macro averaged F1 score, the aggregated model obtained 0.8131 compared with the model trained in a centralized way which obtained a value of 0.9356. The micro F1 score is calculated by summing the true positives, true negatives, and false positives for each of the three classes (Equations (6), (8) and (10)). While in the macro average F1 score, all classes have the same weight, in the micro F1 score all samples have the same weight. Different values for the F1 macro and the F1 micro metrics show that the dataset was imbalanced. While the two presented models perform differently, our proposed method contains three entities isolated one from another, with each entity having a subset from the entire dataset and not all the samples as the centralized model. The minimum available clients for training and evaluation was configured as equal to two, which implies that if one of the deployed FL clients is not ready to perform training or validation, the FL process can proceed with the remaining two clients. This configuration can prevent the entire system to get blocked by the FL clients but, as can be seen in the metrics obtained during training (Table 4), it has an impact on the performance of the global model. In three rounds from the total of ten chosen for training, only two clients were available for training and validation due to hardware limitation. For FedAvg (Equation (1)), k represented the total number of clients who participated in a specific training round and the total was equal to 2. Thus, not involving in the FL training a third client can impact the performance of the global model. However, this can be a real-world scenario as, in some cases, a healthcare institution may not be available for training due to hardware constraints or network latency.

While the three clients are not sharing samples from the dataset, since they are deployed locally, they are sharing the same hardware resources. The training time in FL is significantly greater than performing the training on a single model because the pre-trained weights for each VGG-16 model have to be loaded for each client on the same GPU. In recent years, the conventional method of training DL algorithms has been proven effective in healthcare, especially in medical imaging. The dataset used for training and validation is stored locally on the machine used for training. The model obtained after the training can be then deployed on other machines or devices for predictions. By using this method, an assumption is made: the data which will be used for training can be copied on that specific machine. This can be done if the data belongs only to a medical department or hospital. However, in some cases the data from multiple sources cannot be copied on the same machine and the collaboration between medical institutions or departments can be difficult due to both legally and privacy concerns. Furthermore, in collaboration between these institutions the question regarding which party should process the data will rise. In this paper, we locally deployed three FL entities. Each of these entities had an individual dataset and trained their own local model without having information about every entity involved in the process. Furthermore, lung CT images from different public datasets were used, which can represent a real-world scenario in which multiple healthcare institutions can train a global model on images obtained from different CT scans. Leveraging these FL capabilities can open new path in healthcare research and diagnosis by collaborations between institutions.

**Figure 3:**
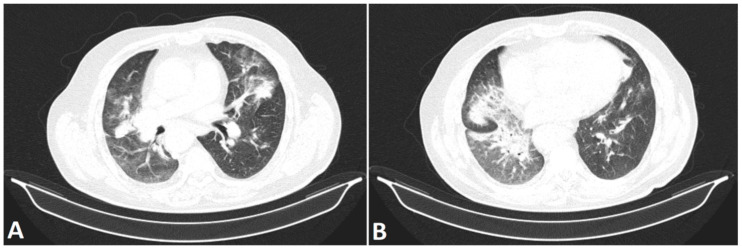
Two different slices (lung window) from the chest CT scan labeled as COVID-19 solely based on the lung changes by the algorithm presented in this paper. The patient had been examined prior to the first officially reported patient to have contracted COVID-19 in Romania. The chest CT scan illustrates bilateral confluent ground-glass opacities mostly distributed in the periphery of the lung (**A**) and a diffusely delimited consolidation area affecting both the middle and the right inferior lobe (**B**).

In Romania, the first reported patient to have contracted COVID-19 was officially declared on 26 February 2020 [68]. However, after connecting the aggregated global model to our internal PACS and running it backward in order to analyze chest CT scans prior to 26 February 2020, the software encountered chest CT changes suggestive of COVID-19 in a patient investigated in our Medical Imaging Department on 28 January 2020. The expert radiologists that labeled the initial dataset agreed the CT aspect could have been associated with a COVID-19 infection at that time. Several images from this patient are illustrated in Figure 3A,B.

## 6. Conclusions

Our paper presents a FL approach with pre-trained models for COVID-19 detection. Based on our results, we recommend using an automated AI software in order to detect COVID-19 based on lung imaging changes as an adjuvant diagnostic method to the current gold standard (RT-PCR) in order to greatly enhance the management of these patients and also limit the spread of the disease, not only to the general population but also to the healthcare professionals. Moreover, in the absence of COVID-19 lung CT changes, our AI-assisted software is capable to identify a normal lung aspect and rule out lung cancer or a non-COVID-19 lung infection.

Since the pandemic started in 2020, there have been numerous attempts to develop DL models capable of identifying COVID-19 based on the chest CT imaging findings. In our proposed method, a pretrained VGG-16 model was used. However, instead of training the model on the entire dataset in a centralized way, three individual clients have been deployed, each client having its own dataset. A client can be represented by a medical institution that has a private dataset. These institutions can collaborate to produce a global model. While FL has open problems such as adversarial attacks, bias in training data, or system-induced bias [57], FL still represents a promising technique for collaboration between institutions to create a DL model while keeping the dataset on the client.

## Figures and Tables

**Table 1:** Multiple AI-based methods using CNNs to detect COVID-19 on chest X-rays.

Paper	Sample Size	Algorithm	Results
Mahmud et al. [18]	305 COVID-19, 1538 Normal aspect, 1493 Viral pneumonia, 3780 Bacterial pneumonia	ConvxNet	ACC: 0.900 Recall: 0.890 Spe: 0.890
Rajaraman et al. [19]	314 COVID-19, 1583 Normal aspect, 3780 Bacterial pneumonia, 1493 Viral pneumonia, 11,002 Varied pneumonia	U-Net, VGG-16, Inception-V3, Xception, DenseNet-121, NasNet-Mobile	ACC: 0.930 Sen: 0.970 Spe: 0.860
Rahimzadeh et al. [20]	180 COVID-19, 6054 Pneumonia, 8851 Normal aspect	ImageNet, Xception, ResNet50	ACC: 0.914
Chowdhurry et al. [21]	423 COVID-19, 1485 Viral pneumonia, 1579 Normal aspect	MobileNetv2, SqueezeNet, ResNet18, ResNet101, DenseNet201, CheXNet, Inceptionv3, VGG19	ACC 0.979 Sen 0.979 Spe 0.988
Vaid et al. [22]	181 COVID-19, 364 Normal aspect	Modified VGG19	ACC: 0.963
Brunese et al. [23]	250 COVID-19, 3520 Normal aspect, 2753 Pneumonia	VGG16	ACC: 0.960 Sen: 0.960 Spe: 0.980
Khan et al. [24]	284 COVID-19, 310 Normal aspect, 320 Bacterial pneumonia, 327 Viral pneumonia	CoroNet	ACC: 0.900 Spe: 0.960 Recall: 0.890
Ismael et al. [25]	180 COVID-19 200 Normal aspect	ResNet18, ResNet50, ResNet101, VGG16, VGG19	ACC: 0.947

**Table 2:** Multiple AI-based methods using CNNs to detect COVID-19 on chest CT scans.

Paper	Sample Size	Algorithm	Results
Ko et al. [28]	3993 Chest CT images COVID-19, Non-COVID-19 pneumonia, Non-pneumonia	VGG16, ResNet-50, Inception-v3, Xception	ResNet-50 ACC: 0.998 Sen: 0.995 Spe: 1.000
Ying et al. [29]	777 COVID-19, 708 Normal aspect, 505 Bacterial pneumonia	VGG16, DenseNet, ResNet, DRE-Net	DRE-Net ACC: 0.94 Recall: 0.93 AUC: 0.99
Wang et al. [30]	5372 Raw chest CT images COVID-19, Other pneumonia	DenseNet COVID-19Net	Training ACC: 0.812 Sen: 0.789 Spe: 0.899 Validation 1 ACC: 0.783 Sen: 0.803 Spe: 0.766
Gozes et al. [31]	157 Chest CT scans COVID-19, Non-COVID-19 aspect	ResNet-50-2	Sen: 0.982 Spe: 0.922
Fu M et al. [32]	60,427 CT scans	ResNet-50	ACC: 0.989 Sen: 0.967 Spe: 0.993

**Table 3:** VGG-16 configuration for the proposed method, adapted from [60].

Layer Id	D Configuration
	16 weight layers
	Input (224 × 244 × 3)
1	conv3-64 conv3-64
	maxpool
2	conv3-128 conv3-128
	maxpool
3	conv3-256 conv3-256 conv3-256
	maxpool
4	conv3-512 conv3-512 conv3-512
	maxpool
5	conv3-512 conv3-512 conv3-512
	maxpool
6	FC-128
7	FC-3
	Softmax

**Table 4:** Performance metrics for the aggregated model and centralized model during the training phase.

Model	Categorical Accuracy	F1_μ_	F1_M_	Cohen’s Kappa Score	Matthews Correlation Coefficient	Training Time (Seconds)
Centralized VGG-16	0.9390	0.9390	0.9356	0.9053	0.9053	998.129
Proposed method—FL VGG-16	0.8382	0.7865	0.8131	0.6816	0.6917	1960.73

**Table 5:** Performance metrics for the aggregated model and centralized model during the validation phase.

Model	Categorical Accuracy	F1_μ_	F1_M_	Cohen’s Kappa Score	Matthews Correlation Coefficient
Centralized VGG-16	0.79	0.79	0.7741	0.6804	0.6856
Proposed method—FL VGG-16	0.7932	0.7865	0.7246	0.6441	0.6894
